# Supplementary material for: Comprehensive Annotation and Functional Exploration of MicroRNAs in Lettuce
Source: Front Plant Sci. 2021 Dec 24;12:781836. doi: 10.3389/fpls.2021.781836 (PMC8739914; doi:10.3389/fpls.2021.781836)
Supplement: Supplementary file 2 [file Data_Sheet_8.ZIP › GSM2230748.plot/Lsa-miRN1655_Lsat_1_v5_gn_5_57381.1_2653_TPlot.pdf]

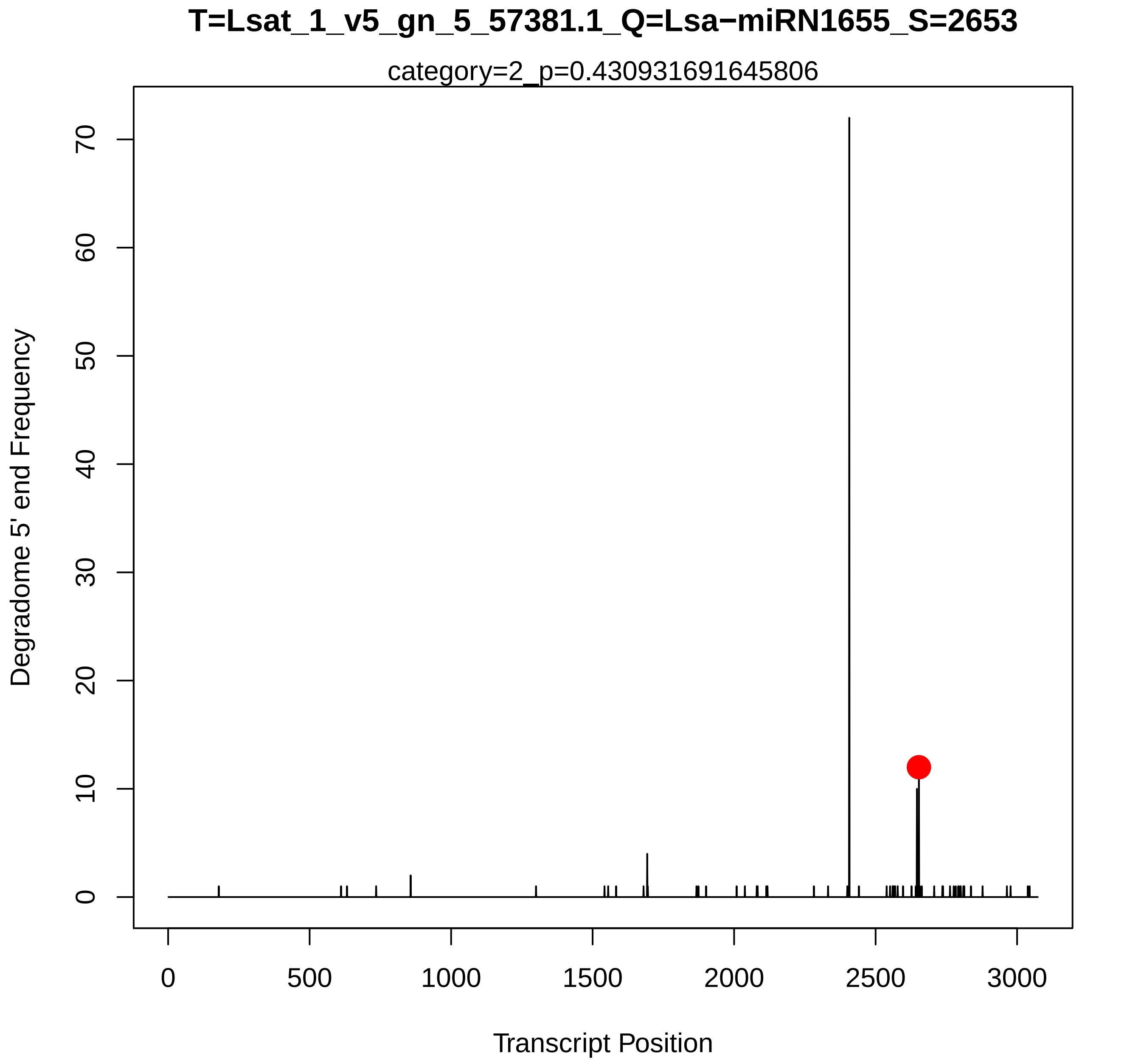
T=Lsat_1_v5_gn_5_57381.1_Q=Lsa−miRN1655_S=2653
category=2_p=0.430931691645806
Degradome 5' end Frequency
0
10
20
30
40
50
60
70
0
500
1000
1500
2000
2500
3000
Transcript Position
